# CRISPR/Cas9 genome editing to create nonhuman primate models for studying stem cell therapies for HIV infection

**DOI:** 10.1186/s12977-022-00604-5

**Published:** 2022-08-10

**Authors:** Jenna Kropp Schmidt, Matthew R. Reynolds, Thaddeus G. Golos, Igor I. Slukvin

**Affiliations:** 1 Wisconsin National Primate Research Center, University of Wisconsin-Madison, Madison, WI USA; 2 Department of Pathobiological Sciences, University of Wisconsin-Madison, Madison, WI USA; 3 Department of Comparative Biosciences, University of Wisconsin-Madison, Madison, WI USA; 4 Department of Obstetrics and Gynecology, University of Wisconsin-Madison, Madison, WI USA; 5 Department of Pathology and Laboratory Medicine, Wisconsin National Primate Research Center, University of Wisconsin-Madison, 1220 Capitol Court, Madison, WI 53715 USA; 6 Department of Cell and Regenerative Biology, University of Wisconsin-Madison, Madison, WI USA

**Keywords:** Nonhuman primates, HIV, SIV, CRISPR/Cas9, CCR5, HSC transplantation, Pluripotent stem cells

## Abstract

Nonhuman primates (NHPs) are well-established basic and translational research models for human immunodeficiency virus (HIV) infections and pathophysiology, hematopoietic stem cell (HSC) transplantation, and assisted reproductive technologies. Recent advances in CRISPR/Cas9 gene editing technologies present opportunities to refine NHP HIV models for investigating genetic factors that affect HIV replication and designing cellular therapies that exploit genetic barriers to HIV infections, including engineering mutations into *CCR5* and conferring resistance to HIV/simian immunodeficiency virus (SIV) infections. In this report, we provide an overview of recent advances and challenges in gene editing NHP embryos and discuss the value of genetically engineered animal models for developing novel stem cell-based therapies for curing HIV.

## Background

Nonhuman primates (NHPs) have been instrumental in advancing our knowledge of HIV pathogenesis, prevention, and therapies [1, 2]. Simian immunodeficiency virus (SIV) and chimeric simian-human immunodeficiency virus (SHIV) infections of NHPs are well-characterized models of HIV infections, faithfully recapitulating key aspects of HIV infections, including the rapid seeding of viral reservoirs, sustained virus replication, the gradual loss of peripheral CD4 + T cells, and the development of simian acquired immunodeficiency syndrome (AIDS) [3–5]. Additionally, NHPs provide several advantages over humans for HIV-cure research. First, antiretroviral therapy (ART)-suppressed NHPs can undergo treatment interruption to measure the time to viral rebound (TTR) to determine intervention efficacy (i.e., more efficacious cure strategies creating longer TTRs), without the ethical concerns of enriching drug-resistant variants. Second, NHPs can be used in terminal studies to systematically measure viral reservoirs in lymphoid and non-lymphoid tissues, identifying tissues that are refractory to treatment and focus new cure strategies to these reservoirs. Third, infecting NHPs with clonal SIV/SHIVs makes it possible to track viral evolution in response to therapeutic pressures, identifying mutations that escape various cure interventions and providing insights into counteracting these viral adaptations.

**Fig. 1:**
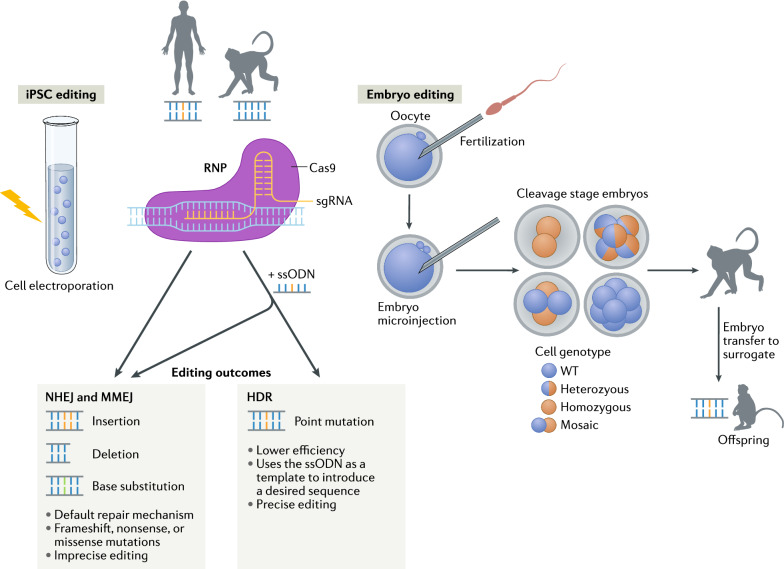
Cell and embryo based genome editing approaches. To introduce a mutation associated with human disease (orange nucleotide pair) into monkey iPSCs and embryos, a Cas9-gRNA ribonucleoprotein complex (RNP) with or without a single-strand oligodeoxynucleotide (ssODN) template containing the desired mutation may be delivered via cell electroporation or microinjection into one-cell embryos. The double-stranded DNA break incurred upon Cas9 cleavage may be repaired preferentially by non-homologous end joining (NHEJ) or alternatively, by homology directed repair (HDR). Repair by canonical NHEJ or an alternative NHEJ pathway via microhomology-mediated end joining (MMEJ) are the cellular default repair mechanisms which often introduce insertions or deletions resulting in gene disruption due to frameshift, nonsense or missense mutations. When provided an ssODN template, repair may occur by HDR to create more precise edits by utilizing the provided template to introduce the desired mutation. Of note, despite co-delivery of an ssODN template, repair by NHEJ will predominate. Upon introducing edits, iPSCs can be differentiated into immune cells and subjected to experimental infection to assess phenotypic and functional responses to validate gene editing strategy and targeted embryos may be transferred to a surrogate to produce edited offspring containing the desired mutation. Abbreviations: iPSC induced pluripotent stem cells, WT wild-type.

The emerging field of genetically modified NHPs can complement HIV studies by editing genes responsible for controlling HIV infections. Here, we review recent progress in using CRISPR/Cas9 methods to edit NHP genomes and how gene-edited NHPs can advance HIV research. We also highlight current progress in editing the *CCR5* gene in NHP embryos and induced pluripotent stem cells (iPSCs), and highlight the value of *CCR5*-edited macaques for developing curative stem cell therapies. Figure 1 provides an overview of iPSC- and embryo-based editing approaches that will be discussed as well as predicted editing outcomes for generating NHPs containing human disease-associated mutations.

### CRISPR/Cas9 technology for introducing mutations

Genome editing by application of CRISPR/Cas9 technology offers promise for creating genetic nonhuman primate models of human disease. The advantage of the CRISPR/Cas9 system over other genome editing systems (i.e. zinc finger nucleases (ZFNs) or transcription activator-like effector nucleases (TALENs)) is the relative ease in design of the guide RNA (gRNA) to target the gene of interest and the ability to readily synthesize Cas9 mRNA or protein for delivery to cells. The CRISPR/Cas9 system is comprised of a gRNA that when complexed with Cas9 endonuclease guides the ribonucleoprotein complex (RNP) to the target site [6, 7]. The Cas9 endonuclease recognizes a protospacer adjacent motif (PAM), or 5′-NGG-3′ sequence, and will cleave the DNA 3 base pairs upstream of the PAM resulting in a double-stranded DNA break [6, 7].

The double-stranded DNA break induced by the Cas9 endonuclease is repaired with intrinsic cellular machinery in one of two ways, either through nonhomologous end joining (NHEJ) or homology directed repair (HDR) [6, 8, 9]. Repair by canonical NHEJ entails the ligation of the ends without utilizing a homologous template serving as a guide. An alternative NHEJ pathway utilizes microhomology of 5–25 base pairs near the cut site to serve as a guide for joining ends and is known as microhomology-mediated end joining (MMEJ) [8]. DNA breaks are more commonly repaired by NHEJ as the repair can occur at any time during the cell cycle, however, this mechanism is error prone and may result in insertions or deletions (INDELS) that are often less than 20 base pairs, and in some instances the sequence may be correctly repaired [8–10]. The introduction of INDELS can create frameshift, missense, or nonsense mutations that may disrupt or knock out gene function. Repair by HDR is much slower in comparison to NHEJ and its occurrence is restricted to the S- or G2-phase of the cell cycle as it requires a template for homologous recombination [8, 9]. To facilitate more precise editing, a single-stranded oligodeoxynucleotide (ssODN) homologous to the target region and containing the edit of interest may be delivered with the CRISPR/Cas9 construct to serve as a homologous template for repair by homologous recombination [8]. NHEJ is considered to be the default repair mechanism for cellular DNA damage [8], thus the efficiency of HDR for creating precise edits is considerably lower in comparison to introducing INDELS.

### Advances and challenges in using CRISPR/Cas9 technologies for generating genetically engineered NHP models

Genome editing by CRISPR/Cas9 in NHP studies has predominantly focused on creating disruption in genes associated with human diseases [11]. Human diseases, however, are often associated with a single point mutation(s) rather than a deletion or disruption of the genetic sequence. Genetic disruptions by targeting DNA with wild-type Cas9 nucleases in NHPs have elucidated the physiological roles of genes in NHPs [12–15], particularly those genes that do not share similar expression patterns between rodents and primates (e.g. *PINK1,* associated with Parkinson’s Disease [14]). In reports of NHP embryo-based genome editing, various targeting approaches have been applied leading to observations that highlight the strengths and limitations of the technology for developing reproducible genetic NHP models of human disease.

The recent CRISPR/Cas9 revolution has impacted NHP research and led to several advances towards creating NHP models of human disease. Table 1 provides an overview of reports of embryo-based genome editing in NHPs with the objective of creating NHP models of human disease by transfer of CRISPR/Cas9 microinjected embryos into surrogates; the table does not include reports describing knock-in sequences or reporters in NHP embryos [16–18]. These studies have yielded a number of important outcomes. First, CRISPR/Cas9 microinjection into NHP embryos has proven successful for targeting single and multiple genes to cause gene disruption [11, 19]. Second, transfer of CRISPR/Cas9 edited embryos into surrogates has led to the birth of live-edited NHPs, albeit the efficiency of obtaining live, edited animals remains low (Table 1; [20]). In addition, edited NHP offspring are predominantly mosaic and it is uncertain that these mutations both genocopy and phenocopy the human disease. Third, genotyping of gonads and gametes has shown that the germline is also edited in offspring derived from CRISPR/Cas9 microinjected embryos, allowing for colony expansion of the mutation [21, 22]. Fourth, several research groups have conducted studies to optimize CRISPR/Cas9 delivery to one-cell fertilized NHP embryos, including testing injection of Cas9 mRNA versus RNP complexes of Cas9 protein and the gRNA [23–25] and varying the concentrations of Cas9 and gRNA [26] or microinjected volume [17], reporting the resulting on-target and off-target genotypes. These studies can serve as a guide for designing NHP CRISPR/Cas9 targeting experiments and have highlighted variables that contribute to the success of early embryo targeting outcomes. Fifth, the demonstration of biallelic editing suggests that creating homozygous NHP mutants is feasible [13, 20, 26, 27]. Finally, a shift in research focus to create heterozygous mutations has led to successful allele-specific targeting. For example, Tsukiyama et al. [28] targeted the paternal allele of the *PKD1* gene in cynomolgus macaque one-cell embryos as most human patients with autosomal dominant polycystic kidney disease are heterozygous for *PKD1* mutations.

**Table 1:** Embryo-based genome editing reports in NHPs with an objective towards creating models of human disease

References	Species	Disease Model	Gene	CRISPR Construct	No. CRISPR/Cas9 microinjected zygotes	No. embryos transferred, surrogates, pregnancies	Offspring Produced	% Offspring Edited	% edited live offspring/ embryos transferred	Notable editing outcomes
Niu et al. [29], Chen et al. [21], Kang et al. [12]	C	Adrenal hypoplasia congenita, hypogonadotropic hypogonadism	DAX1*	5 sgRNAs Cas9 mRNA	186	83 29 10	6 live 13 miscarriage	NS*	NS*	*DAX1* mutations observed in several tissues including testis Germline acquisition of edits reported by Chen et al. 2015
Chen et al. [30], Wang et al. [86]	R	Duchenne muscular dystrophy (DMD)	*DMD*	2 sgRNAs Cas9 mRNA	NS	179 59 17	14 live 4 stillborn 8 miscarriages	61.1	5.03	WGS revealed no off-target editing
Wan et al. [26]	C	Tumorigenesis	*p53*	sgRNA Cas9 mRNA	108	62 13 4	3 live 2 miscarriages	40	3.2	Biallelic editing reported in 1 live infant
Tu et al. [25]	C	NS	*ASPM*	2 sgRNAs Ubi-Cas9 mRNA	NS	178 47 11	6 live	66.7	2.25	A ubiquitin-tagged Cas9 may reduce but not eliminate mosaicism
Zuo et al. [27]	C	Paroxysmal kinesigenic dyskinesia	*PRRT2*	1 or 3 sgRNAs Cas9 mRNA	NS	84 NS NS	6 live 6 stillborn	58.3	5.95	A complete knock-out was produced by targeting with 3 sgRNAs
Zhao et al. [33, Tu et al. [91]	C	Autism Spectrum Disorder	*SHANK3*	2 sgRNAs Cas9 mRNA	NS	116 37 3	1 live 1 stillbirth 1 miscarriage	100	0.86	An 11.5 kb deletion between the two targeted exons of *SHANK3* detected in the miscarried fetus
Zhang et al. [13]	C	*SIRT6*-null	*SIRT6*	sgRNA Cas9 mRNA	98	48 12 4	3 live 1 miscarriage	75	6.25	Biallelic editing observed in three offspring
Yang et al. [14]	R	Parkinson’s Disease	*PINK1*	2 sgRNAs Cas9 mRNA	158	87 28 11	11 live 4 miscarriages	73.3	5.06	A ~ 7.2 kb deletion in *PINK1* detected in three offspring
Zhou et al. [22]	C	Autism Spectrum Disorder	*SHANK3*	2 sgRNAs	NS	178 26 12	9 live 9 deceased	55.5 (of live)	2.80	Three F1 edited heterozygous SHANK3-mutants confirms germline transmission of edits
Qiu et al. [88]	C	circadian-related disorders	*BMAL1*	1–2 sgRNAs Cas9 mRNA	NS	88 31 10	8 live 2 miscarriages	60	6.82	Three live knock-out infants and two mosaic generated with no off-target editing
Tsukiyama et al. [28]	C	Polycystic kidney disease	*PKD1*	sgRNA Cas9 mRNA	403	86 86 29	14 live 8 stillbirths 7 miscarriages	100	16.28	Reported highest proportion of live edited offspring and use of an allele-specific targeting approach
Schmidt et al. [80]	MCM	HIV-resistance	*CCR5*	2 sgRNA Cas9 RNP	NS	50 6 0	0 live	0	0	Whole embryo and blastomere genotyping resulted in 23–37% bilallelic editing of *CCR5*
Chen et al. [92]	C	Parkinson’s Disease	*PINK1*	2 sgRNA Cas9-D10A mRNA	126	51 25 6	4 live	75	5.88	First report of targeted-offspring by Cas9-D10A with an editiing efficiency of 9.1–100%
Wang et al. [42]	C	Hutchinson-Gilford progeria syndrome	*LMNA*	gRNA Base editor mRNA	86	41 11 6	5 live 1 miscarriage	83.3	12.20	First report of live edited offspring generated via base-editors; offspring carried a precise C-T conversion in *LMNA*

Live births indicate those born alive, although infant death may have occurred hours to months after birth

*C* cynomolgus macaque, *MCM* Mauritian cynomolgus macaque, *R* rhesus macaque, *NS* not specified

*Multiplex editing of three genes including *DAX1*; total number of *DAX1*-edited offspring is not clear from the three reports describing these offspring

These pioneering CRISPR/Cas9 studies in NHPs have also revealed several challenges that prevent high throughput production of edited-NHPs to recapitulate human disease phenotypes. While genetic disruption can highlight the physiological roles and impact of a specific gene, disruptions may not produce similar symptoms that arise from a point mutation associated with human disease. This could be attributed at least partially/or in many cases to mosaicism in CRISPR/Cas9 edited animals [22, 24, 28–30], while human patients contain the disease-associated mutation in every cell within the body. Microinjection into mature oocytes at the time of fertilization as shown in human embryos may result in a more uniform editing pattern [31, 32], although this approach remains to be explored in NHPs. Despite transfer of a large number of embryos to many surrogates, relatively few live, edited NHP offspring have been produced [20], thus requiring substantial NHP resources to produce few subjects. Of those edited offspring, a wide range of mosaic genotypes are observed across small cohorts leading to disparity and lack of repetition in molecular and physiological outcomes associated with the mutation. Breeding of founder (F0) animals that carry mosaic edits in the germline may produce a cohort of F1 edited infants with a wide range of editing genotypes. However, both homozygous and heterozygous *CCR5*-edited F1 infants are relevant for modeling human HIV infection because *CCR5∆32* heterozygous individuals have delayed disease progression. Whole genome sequencing (WGS) of DNA from F0 animals and progeny would serve to determine the genotypes of the model animals to better understand genotype to phenotype relationships.

The consequence of undesired on-target editing warrants concern. Yang et al. [15] showed that targeting the *PINK1* gene to induce gene disruption resulted in a large-scale deletion of ~ 7.2 kb that likely contributed to early death and the extent of neuronal loss in deceased newborn monkey brains. Similarly, large deletions have also been observed following CRISPR/Cas9 targeting in another NHP study [33], and mouse and human embryos [32, 34, 35]. Genetic aberrations associated with embryonic genome editing will be expanded below, and is mentioned here to highlight an issue hampering the introduction of precise genetic mutations.

Recognizing that several of the aforementioned challenges hinder disease modeling in NHPs, careful design of the targeting experiment and advances in both CRISPR/Cas9 targeting approaches and assisted reproductive technologies could serve to improve the success rates in obtaining live-edited offspring and generating NHP models that truly reproduce genetic diseases. The NHP species selected for the genetic model needs to have high DNA sequence homology to the human gene of interest and share similar RNA and protein expression profiles. The gRNAs should be tested in cell culture platforms of the NHP species selected to ensure targeting is achieved before transitioning to embryo injections. Although gRNA design tools may take into account SNPs [36], de novo mutations may arise across generations, hence prior to CRISPR/Cas9 experiments the cells or gamete donors should be sequenced to ensure gRNA complementarity. For validation studies, iPSCs provide a unique platform since they can be easily edited and used to generate the desired cell type to assess the impact of the mutation on the disease phenotype in vitro. Microinjection of CRISPR/Cas9 constructs after fertilization at the one-cell stage has been shown to result in mosaic editing [24, 28–30], whereas injections at the time of fertilization in human embryos have resulted in more uniform editing patterns when performing allele-specific targeting of the paternal allele [31, 32]. Concurrent CRISPR/Cas9 targeting of both parental alleles at fertilization remains to be explored in human or NHP embryos, so it is unclear if biallelic editing would occur. Allele-specific targeting has been demonstrated in human and cynomolgus macaque embryos where the gRNA sequence was homologous to a specific parental allele that contained a SNP unique to the targeting allele, allowing for the introduction of heterozygous mutations [28, 31, 32]. Importantly, humans having a heterozygous *CCR5* deletion show delayed HIV progression [37, 38]; thus, creating either homozygous or heterozygous mutations is relevant to understanding HIV resistance in a NHP *CCR5*-edited genetic model.

A strategy to create precise edits is to implement next-generation Cas9 nucleases, such as base or prime editors, that do not introduce double-stranded DNA breaks and rather facilitate single base conversions [39, 40]. Base editors have been introduced into NHP embryos, demonstrating multiplex editing of several genes [41], and the birth of three live homozygous edited monkeys that displayed features of Hutchinson-Gilford progeria syndrome upon introducing a C-T base conversion to the *lamin A/C* gene [42]. Optimization of base-editing technology to obtain a more uniform editing pattern and comprehensive assessment of on- and off-target editing consequences remains to be explored upon NHP embryo microinjection.

### The value of a CCR5-edited NHP model

The chemokine receptor CCR5 binds RANTES (CCL5), MIP1-alpha (CCL3), and MIP1-beta (CCL4) cytokines [43] and plays a role in mounting an inflammatory response to infection. CCR5 is also the predominant co-receptor for most HIV-1 strains, binding to the HIV envelope protein, promoting viral and cell membrane fusion and viral entry into the host cells [44–47]. However, polymorphisms in the *CCR5* gene can disrupt this essential molecular interaction [37, 48–50]. The best characterized *CCR5* allelic variant contains a CCR5∆32 in the coding region of the second extracellular loop, creating a severely truncated molecule that prevents CCR5 expression, thereby disrupting viral entry [37] and making *CCR5*∆32 homozygous individuals resistant to infection with CCR5-tropic strains of HIV [37, 50–52]. The *CCR5*∆32 is the most common in individuals of European descent with heterozygous and homozygous allele frequencies of 10 and 1%, respectively [50, 53]. However, this mutation is rare in African and Indian populations. In African populations, other types of *CCR5* mutations have been identified, including *CCR5*∆24 [48], C101X [54], the R225X mutation which prevents *CCR5* expression and the D2V mutation which decreases binding to HIV [55]. Other multiple polymorphic variations have been described in the *CCR5* gene, however, many of them fail to protect against HIV infection, even if the same mutation caused impaired binding and functional responses to chemokines [54].

Given the critical role of *CCR5* in HIV infection, disrupting CCR5 binding is a core strategy for HIV cure initiatives [56, 57]. Supporting this strategy are allogeneic hematopoietic stem cell transplants (alloHSCTs) with cells from *CCR5∆32* homozygous donors [58]. In two high profile cases, HIV-resistant donor cells replaced recipient immune cells post-transplant, resulting in the rapid depletion of viral reservoirs, enabling the “Berlin” and “London” patients to stop ART without viral rebound, functionally curing them of HIV[59–62]. To date, these transplants are the only medical interventions to eradicate HIV reservoirs, but they are not scalable nor relevant to most people with HIV. Therefore, advancing our understanding of the underlying mechanism(s) that eradicate HIV reservoirs after *CCR5*-mutant alloHSCT transplants will aid the design of more efficacious treatment regimens available to all people with HIV.

To this end, critical questions remain about how the Berlin and London patients alloHSCTs eliminated viral reservoirs and prevented viral rebound. These questions include: Do allogeneic T cells play a significant role in eliminating host leukocytes and eliminating endogenous HIV reservoirs? Are HIV cures only attainable with *CCR5*-mutant cells, or are similar outcomes achievable with allogeneic HSCs expressing functional CCR5 (wild-type)? When is it safe to withdraw ART after *CCR5*-mutant HSCTs? Are *CCR5*-mutant cells broadly protective against infection, or are they only effective against viral strains using CCR5 as a coreceptor (R5-tropic)? Are homozygous *CCR5*-mutant cells needed to cure HIV, or are heterozygous cells also effective? Does *CCR5* heterozygosity influence HIV reservoir size, and does it help shrink viral reservoirs after alloHSCTs? What engraftment thresholds are necessary to achieve significant clinical benefits from *CCR5*-mutant HSCs? What role do pre-transplant conditioning regimens play in eliminating HIV reservoirs? Can nontoxic conditioning regimens still produce HIV cures?

Investigators have sought to determine whether naturally-occurring *CCR5* mutations exist in experimental NHP models. A genetic study by Chen et al. [63] identified a 24-bp deletion in *CCR5* that prevents *CCR5* from being functionally expressed by more than 98% of red-capped mangabeys. However, this study did not find any deletions within the *CCR5* locus of 9 chimpanzees and 35 rhesus macaques. Thus, genetically modified NHPs are highly desirable for modeling *CCR5*-mutant HSC therapies. In contrast to approaches that employ transgenic HSCs, the use of HSCs from *CCR5*-mutant animals eliminates the issues associated with variations in HSC gene editing efficiency and diminished HSC engraftment potential following ex vivo manipulation of CD34 + cells.

Outside of HIV, *CCR5* polymorphisms may affect the inflammatory responses to other infectious agents. CCR5 is predominantly expressed on leukocytes and, upon binding its pro-inflammatory cytokine ligand, enhances the effector functions of these cells and directs them to sites of infection [44]. Thus, the loss of function with the *CCR5∆32* variant may disrupt immune cell activity and inhibit CCR5-mediated inflammation, which can have beneficial or detrimental impacts on disease outcomes. Indeed, human and animal studies have shown that *CCR5* deficiency has a protective effect against diseases caused by viral, bacterial, and parasitic infections, including *Toxoplasma gondii, Mycobacterium tuberculosis*, *Mycoplasma pneumoniae, Streptococcus pneumoniae, Herpes Simplex* Virus, *Hepatitis B* virus, *Trypanosoma cruzi, Cryptococcus neoformans, Chlamydia trachomatis, Listeria*, and *Plasmodium* (reviewed in [49, 64]) and severe SARS-CoV-2 infections [65]. Conversely, *CCR5*∆32 is associated with increased susceptibility to severe infections with flaviviruses West Nile Virus [66–68] and Tickborne encephalitis virus [69, 70]. Access to *CCR5*-mutant NHPs may open unique opportunities to establish NHP models for severe West Nile virus infection and advance the use of NHPs to study unique disease courses or conditions that occur in humans with *CCR5* polymorphisms. For example, CCR5-deficiency is also associated with decreased risk of graft-versus-host-diseases (GVHD) [71], *CCR5*-mutant NHPs can be used to explore the potential of *CCR5* targeting for GVHD therapies. *CCR5* also plays a role in neuroplasticity, learning and memory and can potentially contribute to cognitive deficit caused by HIV infection [72]. Thus, *CCR5*-edited animals could be an integral tool for assessing mechanisms of HIV neurocognitive disorders.

### Editing CCR5 in Mauritian cynomolgus macaque embryos

To facilitate NHP modeling of curative alloHSCT-based HIV therapies, we explored editing the *CCR5* gene in embryos via CRISPR/Cas9 in Mauritian cynomolgus macaques (MCMs), which have limited major histocompatibility complex (MHC) diversity [73, 74], allowing for control of genetic factors during alloHSCTs and quantifying the effect of MHC-matched allogeneic cells on purging SIV reservoirs. To disrupt *CCR5*, we used two gRNAs to target sequences within exon 2, including a 24-bp deletion region known to be essential for expressing functional *CCR5* in NHPs [63]. Previously, we showed that the *CCR5* gene is more efficiently edited in human iPSCs with dual gRNAs than a single gRNA [75]. To confirm that CRISPR/Cas9-targeted *CCR5* edits also protects macaque cells from SIV infection, we generated iPSCs from MCM fibroblasts, edited their *CCR5* locus, and derived T cells and macrophages. We found that T cells and macrophages produced from *CCR5*-edited fibroblast-derived iPSCs did not support replication of the CCR5 T cell-tropic SIVmac239 and macrophage-tropic SIVmac316 simian immunodeficiency viruses, thus validating our *CCR5* editing strategy [76].

The methods for producing in vitro fertilized embryos from rhesus and Chinese cynomolgus macaques (CCMs) are well established [77–79]. However, our studies unexpectedly revealed differences in reproductive biology between Mauritian and Chinese subspecies, requiring further optimization of assisted reproductive methods for MCMs. Applying a CCM ovarian stimulation protocol to MCM oocyte donors produced relatively few mature oocytes upon laparoscopic follicular aspiration (~ 13.4 oocytes, 4.2% mature oocytes). Extending the follicle stimulating hormone treatment to 11–12 days and performing follicle aspiration between 38 and 40 h post-human chorionic gonadotropin treatment improved recovery of mature MCM oocytes (~ 24.3 oocytes, 56% mature oocytes). Additionally, we optimized in vitro culture conditions to support MCM embryo development to the blastocyst stage [80].

Following fertilization of 240 MCM oocytes by intracytoplasmic sperm injection (ICSI), we microinjected oocytes with Cas9 alone (no gRNA) or a RNP complex comprised of Cas9 complexed to the gRNAs (see Fig. 1 for an overview of the embryo editing approach), or were not microinjected and cultured as control. A reduced cleavage rate in *CCR5* RNP injected oocytes was observed compared to unmanipulated non-injected control oocytes (45.8% vs 72.7%, respectively) [80]. Time-lapse imaging showed a tendency for delayed embryo development in Cas9 alone and *CCR5* RNP-microinjected embryos in comparison to control embryos [80]. Heterozygous and homozygous mutations were detected by PCR in 53.3% and 36.7% of 73 *CCR5 *RNP injected embryos, respectively. In addition, analysis of 129 individual blastomeres from 18 embryos showed that 82% were heterozygotes and 23.5% were homozygotes for the *CCR5* deletion. A mosaic genome editing pattern was observed in ~ 50% of the *CCR5*-edited embryos. Thus, by refining the ovarian stimulation and in vitro culture conditions, we obtained for the first time a cohort of mature MCM oocytes, fertilized them in vitro, and efficiently edited them using CRISPR/Cas9, introducing mutations into *CCR5* in more than 50% of embryos. Significant challenges were encountered with transfer of edited embryos to surrogates to achieve pregnancy, signifying the need for better characterizations of menstrual cycle in MCMs to synchronize embryo transfer timing with surrogates’ implantation window.

### Genomic aberrations following embryo editing in primates

**Fig. 2:**
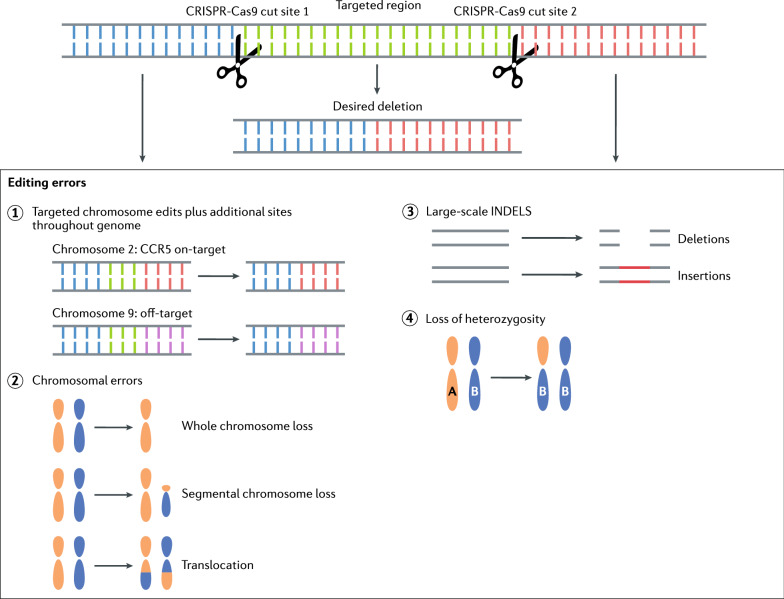
Potential on- and off-target editing outcomes. CRISPR/Cas9 editing can result in both desired on-target editing events and the potential for introducing unexpected gene modifications. Editing errors that may be incurred include off-target edits, whole or segmental chromosome losses and translocations, large-scale insertions and/or deletions (INDELS), and loss of heterozygosity due to a loss of one parental allele and homologous recombination of the retained allele. Editing anomalies can occur at the on-target site as well as at an off-target site(s) that shares homology to the gRNA sequence

A consequence of introducing mutations with CRISPR/Cas9 is the introduction of undesired mutations at the on- and/or off-target sites. Undesired edits introduced at the on-target site by CRISPR/Cas9 include large deletions, translocations and whole or partial chromosome elimination often associated with the formation of micronuclei [10, 32, 34, 81, 82]. Complex chromosomal rearrangements may result in disruption of neighboring genes, chromothripsis and also loss of heterozygosity due to homologous recombination near the target site [35, 81–83]. Figure 2 illustrates potential editing errors that could occur at the on- and/or off-target site(s).

CRISPR/Cas9-associated anomalies at the on-target site have been observed in human and mouse CRISPR/Cas9 microinjected embryos, and include large scale deletions, complete loss of whole and chromosomal segments, and loss of heterozygosity [32, 34, 35, 84]. Embryonic loss of whole chromosomes is particularly a concern as aneuploidy has been associated with implantation failure and miscarriage in humans [85]. Neither the incidence of chromosomal loss and segmentation in CRISPR/Cas9 microinjected embryos, nor the impact of CRISPR/Cas9 induced chromosomal anomalies on developmental trajectory to the blastocyst stage or pregnancy have been extensively explored in NHPs. Off-target analysis following NHP embryo microinjection has focused on WGS and/or cloning PCR amplicons of candidate off-target regions, and no off-target edits with CRISPR/Cas9 have been reported following NHP embryo microinjections [15, 18, 26, 29, 30, 86–88]. However, Zuccaro et al.[32] reported the introduction of segmental losses and indels at off-target sites in human embryos when utilizing an allele-specific targeting approach and microinjection at the time of fertilization. Similarly, WGS analysis of *CCR5*-targeted MCM blastomeres has revealed large-scale deletions at the on-target site that were not previously identified by PCR-based analysis and has also identified off-target edits (Schmidt et al. unpublished, in preparation). Therefore, it is necessary to continue assessing off-target mutations in NHP embryos.

### Success of live edited offspring hinges on advances in assisted reproduction technologies

The greatest hurdle in generating cohorts of live, edited offspring is the lack of efficiency in generating pregnancies from CRISPR/Cas9 microinjected embryos, where advances in assisted reproductive technologies are greatly needed. Assisted reproductive technologies in NHPs are relatively inefficient, where ~ 30% of unmodified in vitro fertilized embryos develop to the blastocyst stage and transfer of cleavage to blastocyst stage embryos results in a pregnancy rate of ~ 30–36% [28, 89]. In the instance of embryonic gene targeting, the target gene may have previously unidentified roles in embryo or fetal development. Hence, it is crucial to know the embryo transfer success rate for a research program’s culture system and breeding colony to identify whether specific mutations result in embryonic lethality. To perform these types of experiments, a substantial pool of regularly cycling females are needed to serve as oocyte donors and/or embryo recipients. In addition, developing embryo cryopreservation strategies would allow for subsequent thaw and transfer of genotyped embryos with confirmed *CCR5* edits.

## Conclusions

The generation of genome edited NHPs will provide a powerful tool to further advance studies of HIV pathogenesis and curative therapies. Studies implementing CRISPR/Cas9 technology to target genes in NHP embryos demonstrate that microinjection of RNPs is sufficient to induce on-target mutations, including *CCR5* mutations rendering cells resistant to SIV. However, across CRISPR/Cas9 genome editing studies in NHPs, the percentage of embryos being transferred resulting in a live, edited NHP offspring ranges from 0 to 16.28% [13, 15, 17, 18, 20, 22, 25–30, 33, 42, 88] and models carrying SIV-resistance mutations are not available yet. Improving embryo culture condition, reducing toxicity of RNPs and frequency of chromosomal aberrations following CRISPR/cas9 editing will be essential to improving NHP model creation. Finally, scientific and ethical considerations, including the selection of proper gene targets, implementation of high animal care standards, and use of validated phenotypic evaluations are also central to ensuring that the created models have translational relevance. Although this review describes advances in *CCR5* editing, CRISPR/Cas9 gene editing in the NHP embryo and iPSCs can be also employed for generating in vitro models and animals with other mutations which affect susceptibilities to HIV, thus facilitating development of highly desirable but currently not available research tools. For example, a recent study knocked out *TRIM5,* a gene encoding a restriction factor that blocks cross-species retrovirus infections, in NHP iPSCs yielding NHP macrophages that are permissive to HIV infection [90], further demonstrating utility of gene editing technologies in advancing NHP models for studies of HIV infections.

## Data Availability

Not applicable.

## Abbreviations

NHP: Nonhuman primates
HIV: Human immunodeficiency virus
HSC: Hematopoietic stem cell
SIV: Simian immunodeficiency virus
SHIV: Simian-human immunodeficiency virus
AIDS: Acquired immunodeficiency syndrome
ART: Antiretroviral therapy
TTR: Time to viral rebound
iPSCs: Induced pluripotent stem cells
alloHSCTs: Allogeneic hematopoietic stem cell transplants
MCMs: Mauritian cynomolgus macaques
CCMs: Chinese cynomolgus macaques
ZFNs: Zinc finger nucleases
TALENs: Transcription activator-like effector nucleases
gRNA: Guide RNA
RNP: Redundant Nibonucleoprotein
PAM: Protospacer adjacent motif
ICSI: Intracytoplasmic sperm injection
WGS: Whole genome sequencing
ssODN: Single-strand oligodeoxynucleotide
NHEJ: Non-homologous end joining
MMEJ: Microhomology-mediated end joining
HDR: Homology directed repair
INDELS: Insertions and deletions
GVHD: Graft-versus-host-diseases
MHC: Major histocompatibility complex
